# Clinical applicability and diagnostic performance of electrocardiographic criteria for left ventricular hypertrophy diagnosis in older adults

**DOI:** 10.1038/s41598-021-91083-9

**Published:** 2021-06-01

**Authors:** Caio de Assis Moura Tavares, Nelson Samesima, Ludhmila Abrahão Hajjar, Lucas C. Godoy, Eduardo Messias Hirano Padrão, Felippe Lazar Neto, Mirella Facin, Wilson Jacob-Filho, Michael E. Farkouh, Carlos Alberto Pastore

**Affiliations:** 1 Instituto do Coracao (InCor), Hospital das Clinicas HCFMUSP, Faculdade de Medicina, Universidade de Sao Paulo, Sao Paulo, Brazil; 2 Peter Munk Cardiac Centre and Heart and Stroke Richard Lewar Centre of Excellence in Cardiovascular Research , University of Toronto, Toronto, ON Canada; 3 Unidade de Eletrocardiografia, Instituto do Coracao (InCor), Hospital das Clinicas HCFMUSP, Faculdade de Medicina , Universidade de Sao Paulo, Av. Dr Enéas de Carvalho Aguiar, 44, Andar AB, Sao Paulo, SP 05403-900 Brazil

**Keywords:** Cardiology, Hypertension

## Abstract

Recently, a new ECG criterion, the Peguero-Lo Presti (PLP), improved overall accuracy in the diagnosis of left ventricular hypertrophy (LVH)—compared to traditional ECG criteria, but with few patients with advanced age. We analyzed patients with older age and examined which ECG criteria would have better overall performance. A total of 592 patients were included (83.1% with hypertension, mean age of 77.5 years) and the PLP criterion was compared against Cornell voltage (CV), Sokolow-Lyon voltage (SL) and Romhilt-Estes criteria (cutoffs of 4 and 5 points, RE4 and RE5, respectively) using LVH defined by the echocardiogram as the gold standard. The PLP had higher AUC than the CV, RE and SL (respectively, 0.70 vs 0.66 vs 0.64 vs 0.67), increased sensitivity compared with the SL, CV and RE5 (respectively, 51.9% [95% CI 45.4–58.3%] vs 28.2% [95% CI 22.6–34.4%], p < 0.0001; vs 35.3% [95% CI 29.2–41.7%], p < 0.0001; vs 44.4% [95% CI 38.0–50.9%], p = 0.042), highest F1 score (58.3%) and net benefit for most of the 20–60% threshold range in the decision curve analysis. Overall, despite the best diagnostic performance in older patients, the PLP criterion cannot rule out LVH consistently but can potentially be used to guide clinical decision for echocardiogram ordering in low-resource settings.

## Introduction

Left ventricular hypertrophy (LVH) is an independent predictor of mortality, and cardiovascular morbidity in hypertensive individuals^1–5^. LVH is a marker of poor prognosis also in elderly patients, although few data are available in this population^6^. The 12-lead ECG is recommended as a universal screening of LVH for patients with hypertension according to international guidelines^7,8^. ECG is accessible worldwide, inexpensive and has established its prognostic capacity^9^. Also, the addition of the ECG-based LVH criteria to common cardiovascular risk scores can increase the prediction performance of such scores^10,11^. However, the diagnosis of LVH by the ECG has some limitations, namely the great number of available criteria^12,13^ and poor sensitivity (4–48%) when compared to echocardiogram and cardiac magnetic resonance imaging (MRI)^13–16^. Additionally, extra cardiac factors can mitigate the depolarization vector (such as body habitus, weight, pericardial fluid, lung disease, lead positioning)^17,18^.

There are limited data regarding ECG sensitivity and specificity for detection of LVH in patients with advanced age^19,20^. This age group will grow in the next years—current estimates are that by 2030 there will be more than 73 million Americans over 65 years^21^ and 20.4 million Brazilians over 70 years^22^—and so will the prevalence of age-related diseases. Recently, a new criterion for LVH detection was proposed^23^—the Peguero Lo-Presti criterion: the sum of the S wave in lead V4 with the deepest S wave in the 12-lead ECG (SD + SV4) with a cutoff of 2.3 mV for female subjects and 2.8 mV for male subjects being considered positive for LVH. The criterion had better AUC than other ECG criteria (Sokolow-Lyon, Cornell voltage, RAvL, RL1). We aim to evaluate the diagnostic performance and clinical applicability of the PLP criterion for LVH detection in older adults, compared to traditional ECG criteria.

## Methods

### Population

**Figure 1:**
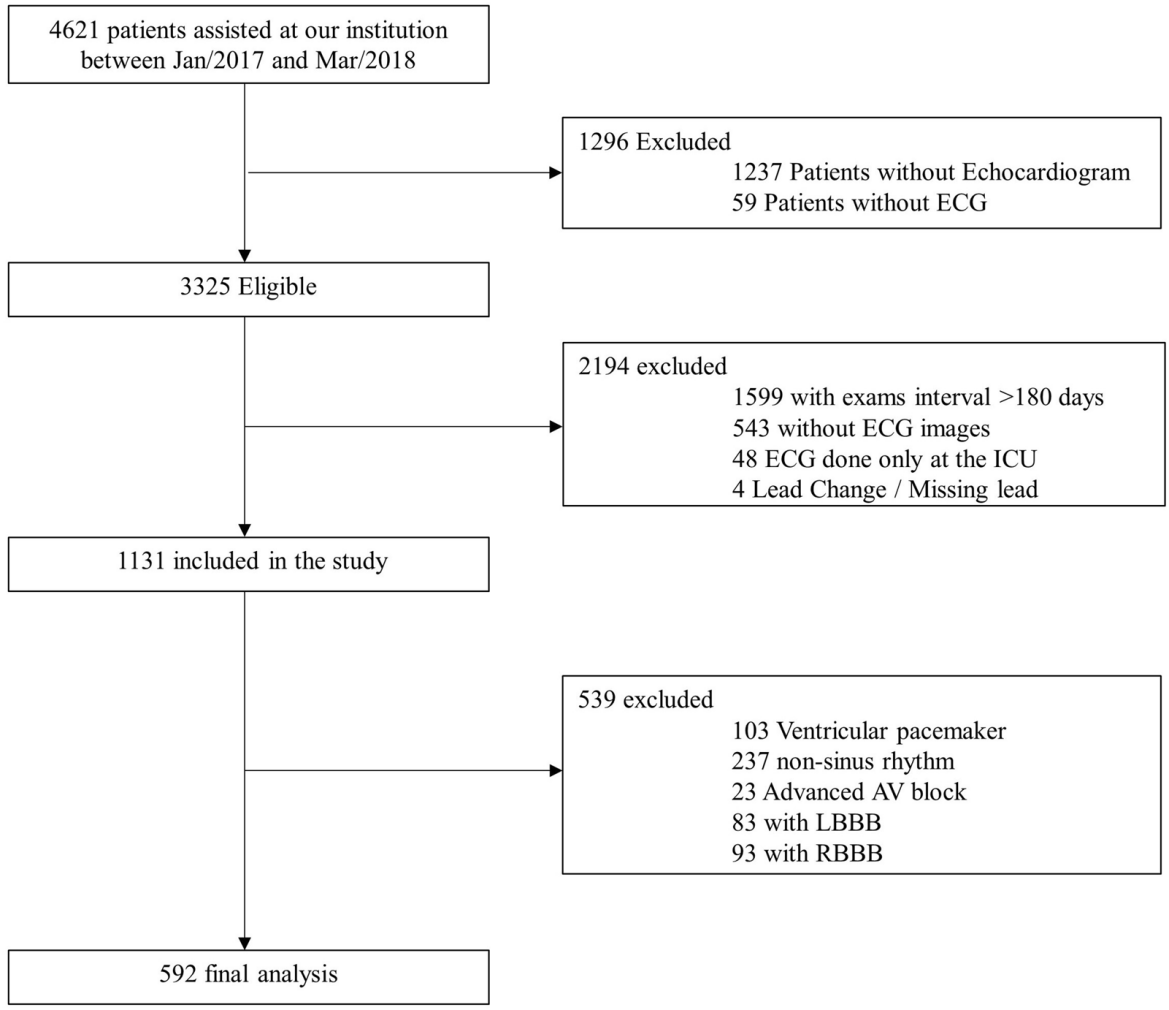
Study flowchart. *ECG* electrocardiography; *ICU* intensive care unit; *LBBB* left bundle branch block; *RBBB* right bundle branch block.

We retrospectively collected data from patients ≥ 70 years old (as of March/31/2018) assisted at our institution—a tertiary care teaching hospital in Sao Paulo, Brazil. From January/2017 to March/2018, all outpatients and inpatients in non-critical units who underwent at least one 12-lead ECG test were evaluated, as outlined in the study flowchart (Fig. 1). All patients with Left Bundle Branch Block (LBBB), Right Bundle Branch Block (RBBB), Atrial Fibrillation/Flutter, Atrial Tachycardia, Supraventricular Tachycardia, Advanced AV Block or Ventricular paced rhythm were excluded from the analysis (see Fig. 1).

### ECG analysis

Standard 12-lead ECGs were acquired at 10 mm/mV calibration and speed of 25 mm/s and all tracings were independently reviewed by two experienced cardiologists from the ECG Unit (NS and MF), blinded to echocardiogram and clinical analysis. In case of discordance, the ECG tracing was reviewed by a third cardiologist (CAMT). Four LVH criteria were calculated from the ECG tracings: Peguero-Lo Presti, Cornel, Sokolow-Lyon, and Romhilt-Estes. The value for the Peguero-Lo Presti criterion was obtained with the sum of the deepest S wave in the tracing plus the S wave amplitude in V4 (SD + SV4), with a cutoff for LVH as described previously^23^: ≥ 2.3 mV for female and ≥ 2.8 for male. The Cornell voltage used a sex-specific voltage criterion as a sum of the R wave in avL plus the S or QS wave in V3 (RavL + SV3) with a cutoff of > 2.0 mV for female and > 2.8 for male^24^. The Sokolow-Lyon was calculated by adding the S wave amplitude in V1 plus the R wave amplitude in V5 or V6, with a cutoff of ≥ 3.5 mV considered positive for LVH^25^. The Romhilt and Estes scoring system was obtained through a sum of 6 characteristics obtained from the ECG: voltage criteria, ST-T abnormalities, left-atrial involvement, QRS axis and duration, intrinsicoid deflection; the point system is summarized in the Supplementary Table S1, available in the supplementary information—for patients with ≥ 4 points the LVH is termed probable and ≥ 5, definite^26^.

### Echocardiography analysis

All echocardiograms were performed at our institution, in accordance with national^27^ and international guidelines^28^. Left Ventricular Mass was calculated using the Devereux formula: left ventricular mass (g) = 0,80 × 1,04 [(septal thickness + internal diameter + posterior wall thickness)^3^ − (internal diameter)^3^] + 0.6 g^29^, and indexed by the Body Surface Area (BSA), calculated by the Dubois Formula (BSA = 0.007184 × height (m)^0.725^ × weight (kg)^0.425^, with LVH defined as > 95 g/m^2^ in females and > 115 g/m^2^ male subjects. Echocardiograms were used as the gold-standard method to diagnose LVH.

### Clinical data

Epidemiological data from all patients were retrieved from the electronic medical record: anthropometric data (height, weight, body mass index), age in years (at the day of echocardiogram exam), comorbidities as diagnosed by the attending physician (hypertension, diabetes, coronary artery disease, prior myocardial infarction, coronary artery by-pass surgery, prior percutaneous coronary intervention, atrial fibrillation, peripheral artery disease, chronic obstructive pulmonary disease), medications prescribed (beta blockers, calcium channel blockers, diuretics, angiotensin-converting enzyme inhibitors (ACEi), angiotensin II receptors blockers (ARB), hydralazine/nitrate). Vital signs were obtained through chart review (blood pressure and heart rate).

### Statistical analysis

Baseline clinical and echocardiographic variables were summarized as mean ± standard deviation for continuous variables and proportions for categorical variables, according to the diagnosis of LVH assessed by the echocardiogram. Continuous variables were compared between groups by means of Student’s t-test or the Wilcoxon rank sum test and categorical variables were compared using the chi-square test.

Sensitivity, specificity and positive and negative predicted values for each ECG criteria were calculated, based on the detection of LVH on the echocardiogram. For comparison between the ECG criteria, we tested for lack of agreement between the tests using the McNemar’s test separately for those with echocardiogram-detected LVH and those without^30^. Receiver operating characteristic (ROC) curves were created by plotting the sensitivity over 1-specificity of each test and the areas under the curves (AUC) were estimated and compared^31^, using the voltage sums for the Peguero-Lo Presti, Sokolow-Lyon and Cornell voltage criteria and the sum of points for the Romhilt and Estes scoring system.

Further performance comparison was done with the F1 score, defined as the harmonic mean of precision (positive predictive value) and recall (sensitivity), ranging from 0 to 100%, with higher scores indicating better model. The F1 score was calculated as F1 = 2*(sensitivity^−1^ + positive predictive value^−1^)^−1^, where positive predictive value is defined as the number of tests correctly identified as positive divided by the total of positives tests^32^. An additional exploratory analysis was carried out to evaluate the diagnostic performance of combined ECG criteria in a stepwise fashion (combining two, three or all ECG criteria).

We used the decision curve analysis framework to incorporate clinical decision making^33^ in our analysis. Following this approach, the net-benefit (NB) for each ECG criteria was calculated by subtracting the proportion of false positives from the true positives, weighted by the relative harm of a false positive and false negative result. Scores were then compared against common strategies of treating all and none of the patients, by subtracting the estimated net-benefit of treating-all strategy from the respective criteria. The resulting net benefit was further used to calculate the number of avoidable interventions (for every 100 patients). Briefly, this method considers how much the physician is willing to treat false positives to avoid not treating a false negative patient. A detailed explanation can be found elsewhere^33^. In our study, the evaluated intervention is ordering an echocardiogram to screen or confirm the diagnosis of LVH. In high resource settings, where echocardiogram is widely available, physicians might tolerate more false positives to avoid missing a true positive (low threshold), whereas in under-resourced settings, the same strategy can lead to waste of scarce resources. Threshold probabilities were selected a priori and chosen to mimic both high (0.1–0.3) and under-resourced (0.3–0.6) theoretical clinical scenarios where elective echocardiogram availability and waiting times are supposed to vary.

We based our manuscript in the 2015 Standards for Reporting of Diagnostic Accuracy Studies (STARD)^34,35^ (Supplementary Table S2, available in the Supplementary Information). A two-sided p < 0.05 was considered statistically significant in all analyses and no adjustment for multiple comparisons was performed. Statistical analyses were performed using STATA version 14.2, Stata Corp LLC^36^ and R software, version 3.6.2.^37^.

### Ethics and consent

The study was approved by the Ethics Committee of the Hospital das Clínicas, Medicine School, University of São Paulo, Brazil (protocol number 3.210.301, project number 08797119.1.0000.0068 on 03/20/2019) and the need for individual signed informed consent was waived. We declare that all methods were performed in accordance with relevant guidelines and regulations.

## Results

### Characteristics of the study population

#### Demographic data

A total of 592 patients were included, 351 without LVH and 241 with LVH, as defined by the echocardiogram. Table 1 summarizes the demographic data of the study population.

**Table 1:** Demographic data.

Demographic data	Non-LVH patients (n = 351)	LVH patients (n = 241)	P value
Age (years)	77.2 ± 5.9	77.9 ± 5.8	0.075
Female	162(46.2%)	139(57.7%)	0.006
BMI (kg/m^2^)	26.3 ± 4.32	26.3 ± 3.9	0.837
SBP (mmHg)	130.5 ± 20.4	135.8 ± 23.4	0.004
DBP (mmHg)	76.0 ± 10.6	75.9 ± 11.9	0.880
Heart rate (bpm)	68.0 ± 14.3	69.2 ± 19.1	0.361
Hypertension	288 (82.1%)	204 (84.7%)	0.408
Type 2 diabetes	118 (33.6%)	95 (39.4%)	0.149
Dyslipidemia	196 (55.8%)	122 (50.6%)	0.211
Paroxysmal atrial fibrillation	62 (17.7%)	35 (14.5%)	0.310
Coronary artery disease	184 (52.4%)	124 (51.5%)	0.817
Previous myocardial infarction	105 (29.9%)	76 (31.5%)	0.674
Previous CABG	58 (16.5%)	45 (18.7%)	0.498
Previous PCI	105 (29.9%)	57 (23.7%)	0.093
Peripheral artery disease	18 (5.1%)	28 (11.6%)	0.004
Chronic obstructive pulmonary disease	30 (8.6%)	32 (13.3%)	0.065
**Medication use**			
ACEi	85 (24.2%)	82 (34.0%)	0.009
ARBs	138 (39.3%)	87 (36.1%)	0.428
CCBs	86 (24.5%)	69 (28.6%)	0.262
Beta blocker	197 (56.1%)	151 (62.7%)	0.113
Hydralazine/nitrate	25 (7.1%)	36 (14.9%)	0.002
Diuretic	140 (39.9%)	143 (59.3%)	< 0.001
Days between echocardiogram and ECG*	7 (0–39)	14 (0–42)	0.269

Demographic data of the cohort, according to the left ventricular hypertrophy status evaluated by echocardiography. Values are mean ± standard deviation or n (%).

*ACEi* angiotensin-converting enzyme inhibitors; *ARBs* angiotensin receptor blockers; *BMI* body mass index; *CABG* coronary artery bypass graft; *CCBs* calcium channel blockers; *DBP* diastolic blood pressure; *PCI* percutaneous coronary intervention; *SBP* systolic blood pressure.

*Median and interquartile rate.

#### Echocardiographic parameters

Patients with LVH had distinctive echocardiographic features: lower ejection fraction, higher mass index, increased diameters (left atrium, interventricular septum, posterior wall, left-ventricular end systolic and diastolic) and relative wall thickness (RWT) and higher prevalence of valvular heart disease (moderate or severe aortic stenosis, aortic regurgitation and mitral regurgitation, p < 0.001 for all comparisons) (available in the Supplementary information, Table S3).

#### Interobserver agreement

Voltage-based criteria had a near perfect agreement as assessed by Cohen’s kappa statistic, with all criteria above 0.90 (Cornell Voltage 0.90, Sokolow-Lyon 0.93 and Peguero-Lo Presti 0.95, all p-values < 0.001). For the Romhilt-Estes scoring system, the agreement between the two observers was moderate (Cohen’s kappa 0.48, p < 0.001).

### Diagnostic performance of the ECG criteria

#### Discriminative power assessed by the area under the curve (AUC)

**Figure 2:**
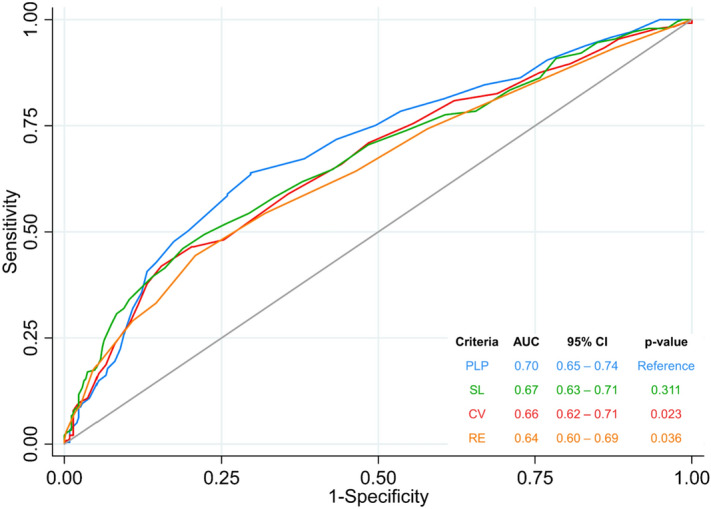
ROC curve and AUC for all ECG evaluated criteria. AUC of the ECG criteria, using the echocardiogram as the reference for LVH. All criteria were compared against the Peguero-Lo Presti criterion. *AUC* area under the curve; *CI* Confidence Interval; *Ref* reference.

The Peguero-Lo Presti criterion had a significantly higher AUC than the Cornell Voltage and Romhilt-Estes criteria (respectively, 0.70 (95% CI 0.65–0.74) vs 0.66 (95% CI 0.62–0.71) vs 0.64 (95% CI 0.60–0.69), respectively, p < 0.05) and a similar AUC compared to the Sokolow-Lyon criterion (AUC = 0.67 (95% CI 0.63–0.71), p = 0.311, Fig. 2).

#### Sensitivity

The Peguero-Lo Presti criterion had higher sensitivity compared to the Sokolow-Lyon voltage (51.9% [95% CI 45.4–58.3%] vs. 28.2% [95% CI 22.6–34.4%]; p < 0.0001), Cornell Voltage (35.3% [95% CI 29.2–41.7%]; p < 0.0001), Romhilt-Estes pointing system with the 5 points cutoff (RE5; 44.4% [95% CI 38–50.9%], p = 0.042) and similar sensitivity compare to the Romhilt-Estes pointing system with the 4 points cutoff (RE4; 54.4% [95% CI 47.8–60.8%], p = 0.497); all analyses using the McNemar’s test for patients with LVH in the echocardiogram.

#### Specificity

The specificity of the Peguero-Lo Presti criterion (82.1% [95% CI 77.6–85.9%]) was inferior to the Cornell Voltage and Sokolow-Lyon criteria (89.7% [95% CI 86.1–92.7%] and 92.6% [95% CI 89.3–95.1%], respectively, with p < 0.0001 for both comparisons using the McNemar’s test for patients without LVH in the echocardiogram. The Peguero-Lo Presti criterion had higher specificity than the RE4 (68.1% [95% CI 62.9–72.9%], p < 0.001) and similar specificity compared to the RE5 (79.2% [95% CI 74.6–83.3%], p = 0.275).

#### Diagnostic performance

The Peguero-Lo Presti had the highest F1 score (58.3%), followed by the Romhilt-Estes 4 points cutoff (54.1%), the Romhilt-Estes 5 points cutoff (50.8%), the Cornell Voltage (47.0%) and finally the Sokolow-Lyon voltage (40.6%). Diagnostic performance of the ECG criteria is summarized in Table 2.

**Table 2:** Diagnostic performance of the ECG criteria.

LVH criteria	Reference: Echocardiogram					Reference: Peguero-Lo Presti	
	Sensitivity (95% CI)	Specificity (95% CI)	PPV (%)	NPV (%)	F1 Score (%)	McNemar test LVH^a^ (comparing sensitivity)	McNemar test no LVH^b^ (comparing specificity)
Sokolow-Lyon voltage	28.2 (22.6–34.4)	92.6 (89.3–95.1)	72.3	65.3	40.6	< 0.0001	< 0.0001
Cornell voltage	35.3 (29.2–41.7)	89.7 (86.1–92.7)	70.2	66.9	47.0	< 0.0001	< 0.0001
Peguero-Lo Presti	51.9 (45.4–58.3)	82.1 (77.6–85.9)	66.5	71.3	58.3	–	–
Romhilt Estes 4 points	54.4 (47.8–60.8)	68.1 (62.9–72.9)	53.9	68.5	54.1	0.497	< 0.001
Romhilt Estes 5 points	44.4 (38.0–50.9)	79.2 (74.6–83.3)	59.4	67.5	50.8	0.042	0.275

Comparison of the performance of the ECG criteria ^a^McNemar test comparing other ECG criteria versus Peguero-Lo Presti in patient with LVH in echocardiogram; ^b^McNemar test comparing other ECG criteria versus Peguero-Lo Presti in patient without LVH in the echocardiogram; ^a^ and ^b^ = p < 0.05 indicates lack of agreement.

*CI* confidence interval; *NPV* negative predictive value; *PPV* positive predictive value.

#### Combination of ECG criteria

The Peguero-Lo Presti criterion also had the highest sensitivity in combination of two (with RE4) or three criteria (with RE4 and Cornell Voltage or RE4 and Sokolow-Lyon) and overall F1 Score (combined with the Sokolow-Lyon and Cornell voltage criteria). Diagnostic performance of combined ECG criteria is summarized in the Supplementary Table S4.

#### Decision curve analysis

**Figure 3:**
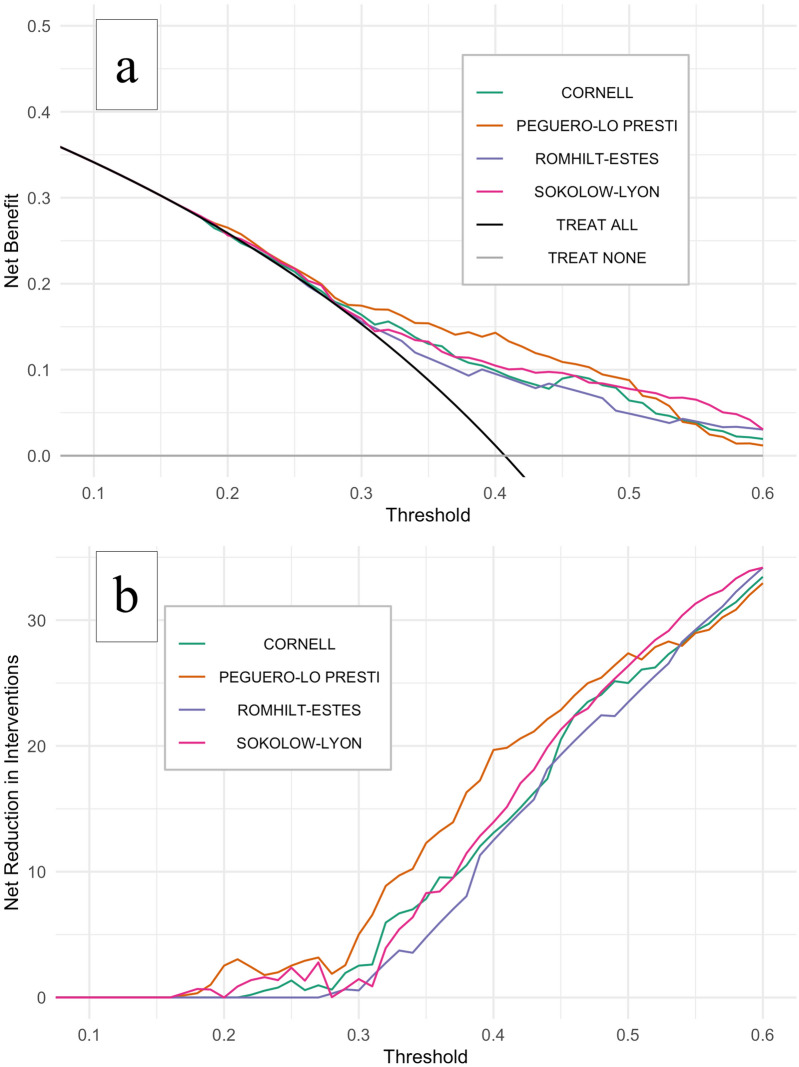
Decision curve analysis for the ECG criteria. Decision curve analysis showing the effect of ECG criteria on the detection of left ventricular hypertrophy as assessed by echocardiogram. Net benefit is plotted against the risk threshold at which the clinician would opt for ordering echocardiogram, compared to strategies of performing echocardiogram to all patients (black line) or none (grey line) (**a**). (**b**) Shows the net reduction in echocardiograms ordered by using different ECG criteria (as shown in the number of unnecessary echocardiograms avoided per 100 patients). Probability threshold range (0.1–0.6) reflect different relative values for harm (performing an echocardiogram in patients without LVH, i.e., false positives) and benefit (identifying a true positive) and were selected a priori to mimic both high (0.1–0.3) and under-resourced (0.3–0.6) theoretical clinical scenarios where elective echocardiogram availability and waiting times are supposed to vary.

The Peguero-Lo Presti criterion had the best net benefit for most 20–60% threshold range as shown in Fig. 3a,b. For thresholds in-between 10 and 20% (low probability threshold scores, applicable to high resource settings, i.e., when physicians might tolerate more false positives ECGs to avoid missing LVH on echocardiogram), we found no/little clinical usefulness for all ECG criteria compared to ordering echocardiograms for all patients. For a probability threshold of 40%(moderate to high probability threshold scores, applicable to under-resourced settings, i.e., when physicians might tolerate fewer false positives to curb ordering of unnecessary echocardiograms) the Peguero-Lo Presti criterion would avoid nearly 20 exams (out of 100 screened patients) when compared to the hypothetical strategy of ordering echocardiogram for all patients. Also, when compared to all other ECG criteria for most 20–60% threshold range, the implementation of the Peguero-Lo Presti criterion would decrease the number of echocardiograms needed to diagnose one patient with echocardiographic LVH (true positive results) without missing additional patients with echocardiographic LVH (false negative results). For illustrative purposes, the impact of screening 100 patients at-risk of LVH with each ECG criterion based on our dataset, using a 40% probability threshold, is pictured in Supplementary Fig. S2 and a direct comparison among all ECG criteria according to varying probability thresholds is summarized in the Supplementary Table S5.

## Discussion

The development of accurate ECG criteria for LVH is an unmet need in Cardiology, especially in the elderly, where very few data are available. In this study, we demonstrated that the PLP had superior performance when compared to the other ECG criteria using the AUC, the F1 score and the decision curve analysis. To the best of our knowledge, we report three new comparisons for the first time: the first between the recently proposed Peguero-Lo Presti criterion and traditional ECG criteria in the elderly, an age group systematic underrepresented in other cohorts. Second, we compared the Peguero-Lo Presti versus the Romhilt-Estes scoring system (that unlike the other criteria, considers more than only the QRS complex voltage for LVH diagnosis). Third, we used the decision curve analysis to help clinicians use a selective strategy to perform echocardiogram in patients at risk of LVH.

After the original publication of the Peguero-Lo Presti criterion several other groups compared the sensitivity of the novel electrocardiographic criterion with other ECG criteria with conflicting results^32,38–45^. The PLP criterion also predicted LVH in another cohort of patients with aortic stenosis^46^ and mortality in a clustered probability sample of the general population^5^. Also, in a cohort of apparently healthy individuals^47^, the specificity of the PLP criterion seemed to be higher in older rather than young individuals. We believe our results add relevant findings: the Peguero-Lo Presti criterion had the highest discriminative performance compared to all other criteria, as assessed by the AUC and the F1 score. We also have shown that, despite previous concerns about its generalizability in certain populations not properly represented in the original publication^39^, the Peguero-Lo Presti criterion can be used in elderly patients with the proposed cut-offs, having an improved sensitivity compared to other voltage criteria and similar sensitivity to the more complex and laborious Rohmilt-Estes scoring system.

Despite the higher sensitivity of the Peguero Lo–Presti criterion, it would not be suitable as a screening test in our population, because near 1 out of 2 patients with LVH would be missed—even in a population from a tertiary center with high prevalence of disease, where sensitivity is probably overestimated due to the spectrum effect^48^. An alternative approach that deserves further exploration to overcome this ECG limitation is to combine different criteria^49,50^, aiming to increase sensitivity and helping surpass the historical inability of the ECG to rule out LVH^51^. Indeed, findings from our exploratory analysis suggests that combination of ECG criteria using the Peguero-Lo Presti criterion increased sensitivity and performance (F1 score) of the ECG.

As the optimal strategy to screen for LVH in patients at risk is still to be determined^7^, the decision curve analysis suggested that the Peguero-Lo Presti criterion might have a role in guiding treatment decisions. The Peguero-Lo Presti provided the best net benefit for most tested thresholds and, compared to other ECG criteria, could optimize the use of echocardiography—a need in low-resource areas, where the waiting time for an elective scheduled echocardiogram can last up to 540 days^52^. As a low-cost, ubiquitous and easily accessible test, the ECG is theoretically the perfect tool for both diagnosis and follow-up of LVH worldwide.

### Study limitations

This was a retrospective single-center study and several limitations bear acknowledgement. First, the gold standard for LVH diagnosis was the two-dimensional echocardiogram that is known to be an operator-dependent test and inferior to MRI^53^. Second, we could not adjust the Sokolow-Lyon voltage product according to body mass index as proposed by Rider and colleagues^54^. Third, since mortality and other long-term cardiovascular endpoints were not available, we could not test the hypothesis that ECG-based LVH assessed by the Peguero-Lo Presti (named electrical LVH) has prognostic implications besides anatomic-based LVH—as both ECG-LVH and echocardiographic LVH may provide prognostic information^55^. Fourth, because we excluded patients with bundle branch blocks, pacemaker and atrial fibrillation, our findings cannot be extrapolated to these groups. Fifth, even though Brazil is a highly ethnically diverse country, ethnic background was not routinely accessed and extrapolations of our findings to certain populations must be done with caution. Finally, our population is representative of a tertiary center with a high burden of cardiovascular disease, which may limit generalizability to the general population. Despite these limitations, we consider that our method is aligned with current clinical practice, where echocardiogram is the most frequently method to assess for LVH and the ECG criteria is rarely adjusted for body habitus. Also, our methodology was very similar to the original publication of the Peguero-Lo Presti criterion^23^.

## Conclusion

Compared to other ECG criteria, the Peguero-Lo Presti criterion had the best diagnostic performance in elderly patients and can potentially be used to guide a selective approach to echocardiogram ordering in low-resource settings. The sensitivity of this criterion, however, remains low and far from what would be expected as a screening tool. Further investigation—possibly by combining different ECG criteria—is needed to fill this long-standing knowledge gap in Cardiology, especially in patients with advanced age, systematically excluded and underrepresented in clinical research.

## Supplementary Information

Supplementary Information.

## Data Availability

The datasets generated and analyzed during the current study are available from the corresponding author on reasonable request.

## Abbreviations

AUC: Area under the curve
CI: Confidence interval
CV: Cornell voltage criterion
ECG: Electrocardiogram
LVH: Left ventricular hypertrophy
PLP: Peguero-Lo Presti criterion
RE: Romhilt-Estes criterion
SL: Sokolow-Lyon voltage criterion
