# Supplementary material for: Clinical applicability and diagnostic performance of electrocardiographic criteria for left ventricular hypertrophy diagnosis in older adults
Source: Sci Rep. 2021 Jun 1;11:11516. doi: 10.1038/s41598-021-91083-9 (PMC8169892; doi:10.1038/s41598-021-91083-9)
Supplement: Supplementary file 1 — Supplementary Information. [file 41598_2021_91083_MOESM1_ESM.docx]

**Supplementary Information**

**Supplementary Table S1**. The Romhilt and Estes Scoring System……………………………………………...2

**Supplementary Table S2**. The STARD 2015 checklist ………………………………………………………....3

**Supplementary Table S3**. Echocardiographic parameters……………………………………………………….5

**Supplementary Table S4**. Diagnostic performance of combined ECG Criteria…………………………………6

**Supplementary Table S5.** Direct comparisons of the ECG criteria using different probability thresholds……..7

**Supplementary Figure 1.** Impact of screening 100 patients at-risk of LVH with each ECG criterion………….8

**Supplementary Table S1**. The Romhilt and Estes Scoring System.

| 1. **Voltage Criteria** | R or S wave in limb leads ≥ 2mV | 3 Points |
| --- | --- | --- |
|  | S wave in V1, V2 or V3 ≥3mV |  |
|  | R wave in V4, V5 ou V6 ≥3mV |  |
| 1. **ST/T morphology with strain pattern** | Without digitalis | 3 Points |
|  | With digitalis | 1 Point |
| 1. **QRS axis deviation ≥ -30º** | 2 Points | |
| 1. **QRS duration ≥0.09seg** | 1 Point | |
| 1. **Left Atrial Abnormality** | P terminal force in V1≥0.1mV and duration ≥40msec | 3 Points |
| 1. **Intrinsicoid Deflection in V5 or V6 ≥ 40msec** | 1 Point | |

Sum of points used for calculation of the Romhilt-Estes criterion. Intrinsicoid Deflection (or R-wave peak time) is measured from the onset of the QRS complex to the peak of the R wave). A value of 4 points is termed LVH as probable and ≥5 points as definite.

**Supplementary Table S2**. The STARD 2015 checklist

|  | **Section & Topic** | **No** | **Item** | **Reported on page #** |
| --- | --- | --- | --- | --- |
|  |  |  |  |  |
|  | **TITLE OR ABSTRACT** |  |  |  |
|  |  | **1** | Identification as a study of diagnostic accuracy using at least one measure of accuracy  (such as sensitivity, specificity, predictive values, or AUC) | 1 |
|  | **ABSTRACT** |  |  |  |
|  |  | **2** | Structured summary of study design, methods, results, and conclusions  (for specific guidance, see STARD for Abstracts) | 1 |
|  | **INTRODUCTION** |  |  |  |
|  |  | **3** | Scientific and clinical background, including the intended use and clinical role of the index test | 2-3 |
|  |  | **4** | Study objectives and hypotheses | 3 |
|  | **METHODS** |  |  |  |
|  | *Study design* | **5** | Whether data collection was planned before the index test and reference standard  were performed (prospective study) or after (retrospective study) | 4-6 |
|  | *Participants* | **6** | Eligibility criteria | 4 and Figure 1 |
|  |  | **7** | On what basis potentially eligible participants were identified  (such as symptoms, results from previous tests, inclusion in registry) | 4 |
|  |  | **8** | Where and when potentially eligible participants were identified (setting, location and dates) | 4 |
|  |  | **9** | Whether participants formed a consecutive, random or convenience series | 4 |
|  | *Test methods* | **10a** | Index test, in sufficient detail to allow replication | 6 |
|  |  | **10b** | Reference standard, in sufficient detail to allow replication | 6 |
|  |  | **11** | Rationale for choosing the reference standard (if alternatives exist) | 6 |
|  |  | **12a** | Definition of and rationale for test positivity cut-offs or result categories  of the index test, distinguishing pre-specified from exploratory | 4 |
|  |  | **12b** | Definition of and rationale for test positivity cut-offs or result categories  of the reference standard, distinguishing pre-specified from exploratory | 4 |
|  |  | **13a** | Whether clinical information and reference standard results were available  to the performers/readers of the index test | 4 |
|  |  | **13b** | Whether clinical information and index test results were available  to the assessors of the reference standard | 4 |
|  | *Analysis* | **14** | Methods for estimating or comparing measures of diagnostic accuracy | 6 |
|  |  | **15** | How indeterminate index test or reference standard results were handled | 6 |

|  | **Section & Topic** | **No** | **Item** | **Reported on page #** |
| --- | --- | --- | --- | --- |
|  |  | **16** | How missing data on the index test and reference standard were handled | 6-7 |
|  |  | **17** | Any analyses of variability in diagnostic accuracy, distinguishing pre-specified from exploratory | 6-7 |
|  |  | **18** | Intended sample size and how it was determined | N/A |
|  | **RESULTS** |  |  |  |
|  | *Participants* | **19** | Flow of participants, using a diagram | Figure 1 |
|  |  | **20** | Baseline demographic and clinical characteristics of participants | Table 1 |
|  |  | **21a** | Distribution of severity of disease in those with the target condition | Table 1 |
|  |  | **21b** | Distribution of alternative diagnoses in those without the target condition | Table 1 |
|  |  | **22** | Time interval and any clinical interventions between index test and reference standard | Table 1 |
|  | ***Test results*** | **23** | Cross tabulation of the index test results (or their distribution)  by the results of the reference standard | Table 3 |
|  |  | **24** | Estimates of diagnostic accuracy and their precision (such as 95% confidence intervals) | Table 3 |
|  |  | **25** | Any adverse events from performing the index test or the reference standard | N/A |
|  | **DISCUSSION** |  |  |  |
|  |  | **26** | Study limitations, including sources of potential bias, statistical uncertainty, and generalisability | 11-12 |
|  |  | **27** | Implications for practice, including the intended use and clinical role of the index test | 10-12 |
|  | **OTHER INFORMATION** |  |  |  |
|  |  | **28** | Registration number and name of registry | N/A |
|  |  | **29** | Where the full study protocol can be accessed | N/A |
|  |  | **30** | Sources of funding and other support; role of funders | 1 |
|  |  |  |  |  |

Abbreviations: **N/A** = not applicable

**Supplementary Table S3**. Echocardiographic parameters

| Echocardiographic parameters | Non-LVH patients  (n=351) | LVH patients  (n=241) | P value |
| --- | --- | --- | --- |
| Ejection fraction (%) | 60.59 ±8.54 | 54.88 ±13.07 | <0.001 |
| Left ventricular mass index (g/m^2^) | 84.10 ± 14.95 | 130.45 ± 26.11 | <0.001 |
| RWT | 0.40 ±0.06 | 0.43 ±0.10 | <0.001 |
| Left Atrium diameter (mm) | 39.14 ± 5.87 | 43.10 ± 5.98 | <0.001 |
| Interventricular septal diameter (mm) | 9.45 ±1.27 | 11.34 ±1.81 | <0.001 |
| Posterior wall diameter (mm) | 8.96 ±1.11 | 10.51 ±1.48 | <0.001 |
| Left ventricular end- diastolic diameter (mm) | 46.92 ±5.19 | 52.70 ±7.16 | <0.001 |
| Left ventricular end-systolic diameter (mm) | 31.33 ±5.13 | 36.95 ±8.42 | <0.001 |
| Moderate or severe aortic stenosis | 23 (6.6%) | 59 (24.5%) | <0.001 |
| Moderate or severe mitral regurgitation | 34 (9.7%) | 47 (19.5%) | 0.001 |
| Moderate or severe aortic regurgitation | 4 (1.1%) | 31 (12.9%) | <0.001 |

Echocardiographic parameters of cohort, according to the left ventricular hypertrophy status evaluated by echocardiography. Values are mean ±standard deviation or n (%). Abbreviations: **RWT** = Relative wall thickness (no unit).

**Supplementary Table S4**. Diagnostic performance of combined ECG Criteria

| ECG combination criteria | | Sensitivity (95% CI) | Specificity (95% CI) | PPV (%) | NPV (%) | F1 Score (%) |
| --- | --- | --- | --- | --- | --- | --- |
| Any  two criteria | CV / SL | 50.2 (44.0-56.4) | 84.9 (81.2-88.9) | 69.5 | 71.3 | 58.3 |
|  | CV / PLP | 55.2 (49.2-61.4) | 80.1(75.8-84.2) | 65.5 | 72.2 | 59.9 |
|  | CV / RE4 | 64.3 (57.7-70.5) | 65.5 (60.4-70.6) | 56.2 | 72.8 | 60.0 |
|  | CV / RE5 | 58.5 (51.8-64.7) | 74.4 (69.5-78.9) | 61.0 | 72.3 | 59.8 |
|  | SL / PLP | 60.6 (54.1-66.8) | 78.6 (74.2-83.2) | 66.1 | 74.4 | 63.2 |
|  | SL / RE4 | 58.5 (52.3-64.7) | 65.2 (60.4-70.4) | 53.6 | 69.6 | 56.0 |
|  | SL / RE5 | 50.2 (44.1-56.2) | 75.8 (71.2-80.2) | 58.7 | 68.9 | 54.1 |
|  | PLP / RE4 | 69.3 (63.5-75.2) | 60.4 (55.0-65.6) | 54.6 | 74.1 | 61.1 |
|  | PLP / RE5 | 64.3 (57.9-70.2) | 68.7 (65.2-73.7) | 58.5 | 73.7 | 61.3 |
| Any  three criteria | CV / SL / PLP | 63.5 (57.3-69.2) | 76.6 (72.2-80.9) | 65.1 | 75.4 | 64.3 |
|  | CV / SL / RE4 | 67.2 (61.1-73.8) | 63.0 (57.8-68.2) | 55.5 | 73.7 | 60.8 |
|  | CV / SL / RE5 | 63.1 (56.5-69.1) | 71.5 (66.7-75.9) | 60.3 | 73.8 | 61.7 |
|  | CV / PLP / RE4 | 71.4 (65.5-77.0) | 59.3 (54.2-64.6) | 54.6 | 75.1 | 61.9 |
|  | CV / PLP / RE5 | 67.2 (61.5-72.7) | 67.0 (62.0-71.9) | 58.3 | 74.8 | 62.4 |
|  | SL / PLP / RE4 | 71.4 (65.0-76.8) | 58.7 (53.3-64.1) | 54.3 | 74.9 | 61.7 |
|  | SL / PLP / RE5 | 67.2 (61.2-73.3) | 66.7 (62.0-71.2) | 58.1 | 74.8 | 62.3 |
| Any criteria | | 73.4 (67.6-78.7) | 57.6 (52.4-62.7) | 54.3 | 75.9 | 62.3 |

Comparison of the diagnostic performance of combined ECG criteria. Abbreviations: **CI** = Confidence Interval; **CV** = Cornell Voltage; **PLP** = Peguero-Lo Presti; **RE4** = Romhilt-Estes 4-points cutoff; **RE5** = Romhilt-Estes 5-points cutoff; **SL** = Sokolow-Lyon; **NPV** = Negative Predictive Value; **PPV** = Positive Predictive Value.

**Supplementary Table S5**. Direct comparisons of the ECG criteria using different probability thresholds.

| ECG Criteria | Probability Threshold | Echocardiogram | | Echocardiographic LVH | |
| --- | --- | --- | --- | --- | --- |
|  |  | Performed | Not performed | Recognized | Missed |
| Peguero-Lo Presti | 10% | 1000 | 0 | 407 | 0 |
|  | 20% | 975 | 25 | 407 | 0 |
|  | 30% | 742 | 276 | 344 | 63 |
|  | 40% | 435 | 565 | 260 | 147 |
|  | 50% | 243 | 757 | 165 | 242 |
|  | 60% | 113 | 887 | 73 | 334 |
| Sokolow-Lyon | 10% | 1000 | 0 | 407 | 0 |
|  | 20% | 968 | 32 | 399 | 8 |
|  | 30% | 760 | 240 | 339 | 68 |
|  | 40% | 434 | 566 | 236 | 171 |
|  | 50% | 220 | 780 | 149 | 258 |
|  | 60% | 115 | 885 | 81 | 326 |
| Romhilt-Estes | 10% | 1000 | 0 | 407 | 0 |
|  | 20% | 1000 | 0 | 407 | 0 |
|  | 30% | 645 | 355 | 302 | 105 |
|  | 40% | 410 | 590 | 222 | 185 |
|  | 50% | 221 | 779 | 135 | 272 |
|  | 60% | 98 | 902 | 71 | 336 |
| Cornell Voltage | 10% | 1000 | 0 | 407 | 0 |
|  | 20% | 988 | 12 | 404 | 3 |
|  | 30% | 806 | 194 | 357 | 50 |
|  | 40% | 451 | 549 | 239 | 168 |
|  | 50% | 203 | 797 | 133 | 274 |
|  | 60% | 100 | 900 | 67 | 340 |

Numbers are given per 1000 patients screened with each ECG criterion. For each probability threshold and ECG criteria, numbers of patients that would perform or not an echocardiogram if the ECG criterion was positive are shown (Echocardiogram column), calculated according to varying thresholds. Also, numbers of true positives (LVH confirmed by the echocardiogram) and false negatives (echocardiographic LVH missed by the ECG) are shown according to ECG criteria and probability thresholds. Probability thresholds were selected *a priori* to mimic both high (0.1-0.3) and under-resourced (0.3-0.6) theoretical clinical scenarios where elective echocardiogram availability and waiting times are supposed to vary. Abbreviations: **ECG**: Electrocardiogram; **LVH** = Left Ventricular Hypertrophy.

**Supplementary Figure 1.** Impact of screening 100 patients at-risk of LVH with each ECG criterion


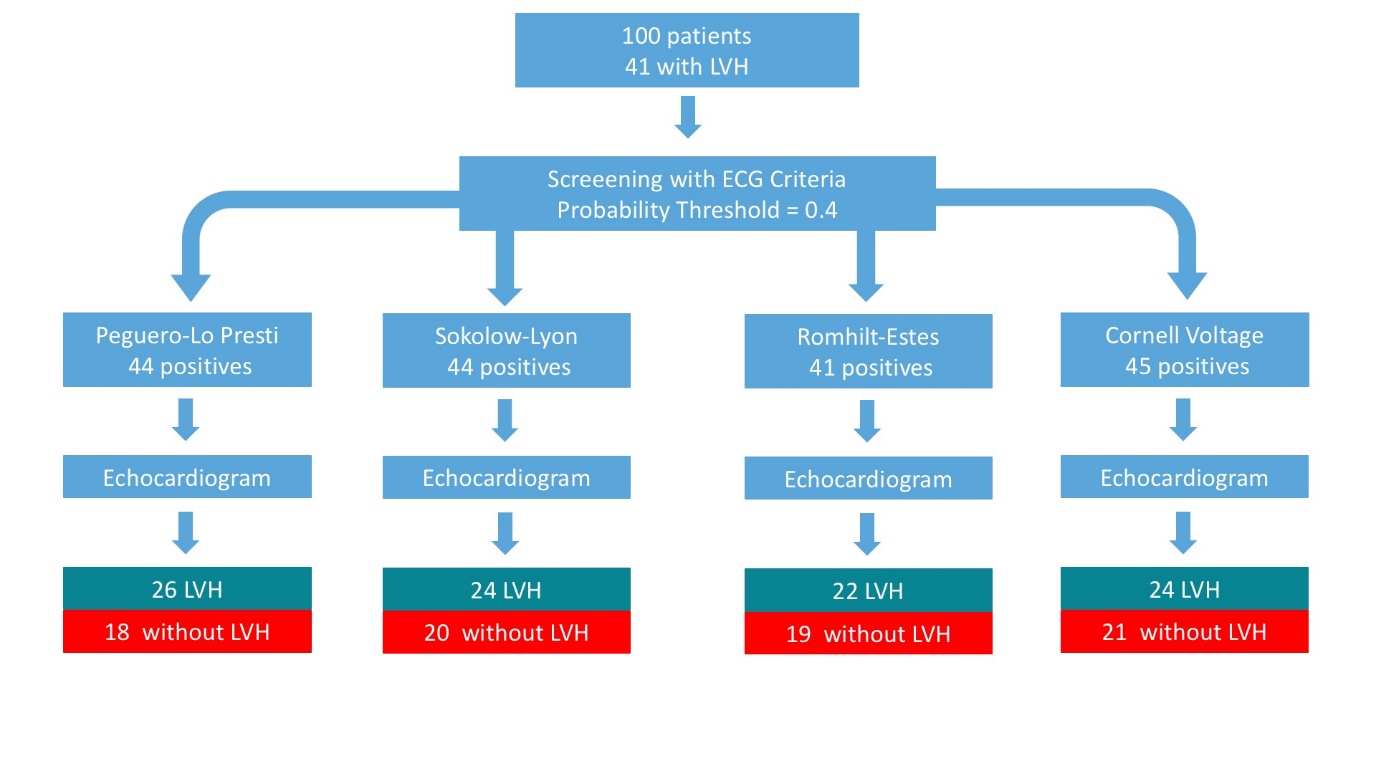


Step by step illustrative impact of screening 100 patients using a 40% probability threshold. First, 100 patients (41 with LVH) are screened with each ECG criterion. If the ECG criterion is positive, then an echocardiogram is performed to confirm or rebut the ECG finding. After the echocardiogram, patients are classified in true positives (echocardiographic LVH) or false positives (without echocardiographic LVH). Abbreviations: **ECG**: Electrocardiogram; **LVH** = Left Ventricular Hypertrophy.
